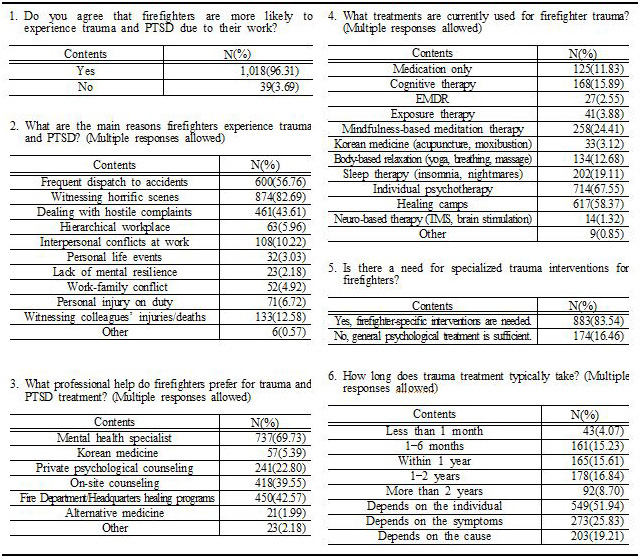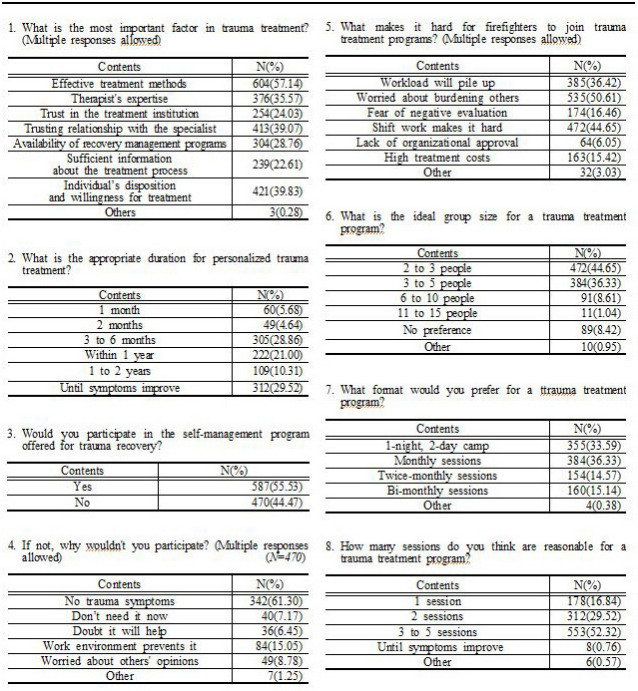# 941 Needs Analysis for the Development of a Hospital-Based Trauma Treatment Protocol for Firefighters

**DOI:** 10.1093/jbcr/iraf019.472

**Published:** 2025-04-01

**Authors:** Se Hee Hwang, Mira Hur, Jeong-Eun Lee

**Affiliations:** Hangang Sacred Heart Hospital of Hallym University Medical Center; Korean Medicine Research Institute, Daejeon University; Department of Medicine, Cha University

## Abstract

**Introduction:**

This study aims to provide foundational data for developing a hospital-based trauma treatment protocol for firefighters by analyzing the perceptions, treatment experiences, self-care practices, and trauma-related needs of 1,057 Seoul firefighters.

**Methods:**

This study collected a total of 1,061 responses through an on-site survey of firefighters who participated in the ‘2023 Seoul Safety Festival’ from May 11 to 13, 2023, and an online survey conducted from May 11 to 31. Ultimately, data from 1,057 firefighters in Seoul, excluding 4 firefighters working outside of Seoul, were used for the analysis.

**Results:**

The results show that 96.31% of respondents recognized a high risk of trauma, such as PTSD, due to the nature of their job, and 45.13% reported experiencing trauma related to their firefighting duties. However, only 25.79% had received professional treatment. Additionally, 55.53% expressed a willingness to participate in intensive treatment programs, and 83.54% of respondents indicated a need for tailored treatment interventions specific to firefighters. These respondents preferred personalized treatment involving mental health specialists, with a duration of 3 to 6 months.

**Conclusions:**

This study provides important recommendations for developing treatment programs that reflect the trauma experiences and perceptions of firefighters, and it is expected to contribute to improving their mental health and well-being.

**Applicability of Research to Practice:**

The results of this study emphasize the need for the development of a trauma treatment program optimized for firefighters and an institutional environment to support it, and are significant in providing basic data for effective interventions in the mental health management and trauma treatment of firefighters in the future.

**Funding for the Study:**

N/A